# The Effect of a Common Daily Schedule on Human Circadian Rhythms During the Polar Day in Svalbard: A Field Study

**DOI:** 10.5334/jcr.186

**Published:** 2019-10-09

**Authors:** Kamila Weissová, Jitka Škrabalová, Kateřina Skálová, Zdeňka Bendová, Jana Kopřivová

**Affiliations:** 1 National Institute of Mental Health, CZ; 2 Faculty of Science, Charles University, CZ; 3 Third Faculty of Medicine, Charles University, CZ

**Keywords:** circadian system, arctic, polar day, social cues, human chronobiology

## Abstract

All Arctic visitors have to deal with extreme conditions, including a constant high light intensity during the summer season or constant darkness during winter. The light/dark cycle serves as the most potent synchronizing signal for the biological clock, and any Arctic visitor attending those regions during winter or summer would struggle with the absence of those entraining signals. However, the inner clock can be synchronized by other zeitgebers such as physical activity, food intake, or social interactions. Here, we investigated the effect of the polar day on the circadian clock of 10 researchers attending the polar base station in the Svalbard region during the summer season. The data collected in Svalbard was compared with data obtained just before leaving for the expedition (in the Czech Republic 49.8175°N, 15.4730°E). To determine the circadian functions, we monitored activity/rest rhythm with wrist actigraphy followed by sleep diaries, melatonin rhythm in saliva, and clock gene expression (*Per1, Bmal1, and Nr1D1*) in buccal mucosa samples. Our data shows that the two-week stay in Svalbard delayed melatonin onset but did not affect its rhythmic secretion, and delayed the activity/rest rhythm. Furthermore, the clock gene expression displayed a higher amplitude in Svalbard compared to the amplitude detected in the Czech Republic. We hypothesize that the common daily schedule at the Svalbard expedition strengthens circadian rhythmicity even in conditions of compromised light/dark cycles. To our knowledge, this is the first study to demonstrate peripheral clock gene expression during a polar expedition.

## 1. Introduction

The regular light/dark cycle has a crucial impact on our circadian clock. The natural daylight period varies between the seasons and its length is also specific to the latitude. In the Czech Republic (49.8175°N), the light/dark cycle during the summer is about 16 hours of light and 8 hours of darkness, and during the winter the light/dark cycle is 8 hours of light and 16 hours of darkness. In Svalbard (77.8750°N), polar days during which the sun is up for 24 h start on the 19^th^ of April and end by the 23^rd^ of August. The long polar night begins on the 11^th^ of November and ends on the 30^th^ of January. Svalbard visitors are thus exposed to extreme light conditions that affect their circadian clocks. Previous studies already reported circadian, sleep or mood disturbances due to constant illumination or constant darkness in a polar environment [1,2,3,4,5]. The aim of our study was to complete this data with the evaluation of clock gene expression in peripheral tissues and with the assessment of changes in the circadian rhythm within the same subjects before departure and after a two-week adaptation period in Svalbard.

## 2. Materials and Methods

### 2.1. Participants

The participants were recruited from a group of Czech researchers attending a 14-day summer expedition in Svalbard. Ten subjects were enrolled in this study (five males and five females) of average age 34.6 ± 8.5 SD. Potential participants were excluded for: 1) having changed time zones during the study, 2) having worked night shifts up to one month before the beginning of the study, or 3) being on any medication that was known to affect their sleep.

Before the beginning of the study, the participants were informed about the underlying scientific principles and the experimental design of the study during an instructional meeting. This included information regarding proper sample storage and collection and the importance of avoiding artificial light exposure during the night periods. Each subject received a prepared kit with nine marked sampling tubes for saliva (opaque tubes) and 14 tubes for buccal scrubs containing RNA stabilizing solution, cytological brushes, and an actigraph device.

All participants signed an informed consent waiver that was in agreement with the Declaration of Helsinki and was approved by the Ethics Committee of the Third Faculty of Medicine of Charles University.

### 2.2. Protocol of the study and sample collection

The study took place over two phases, using the same protocol: The first was in the Czech Republic before departure, and the second part took place in Svalbard during the last day of the participants’ two-week stay.

#### 2.2.1. Czech Republic sampling conditions

The sleep/wake cycle was recorded using the MotionWatch device supplemented with sleep diaries from 10–14 days before departure to the expedition. All subjects were instructed to keep their regular life/work schedules for the entire duration of the measurement period. The saliva and buccal scrub samples were collected 1–3 days before leaving for Svalbard. The sampling started at 7 AM, and all the samples were collected in 4 h intervals over a 24 h period. Two additional times, 9 AM and 9 PM, were inserted into the saliva sampling schedule for more precise estimation of melatonin rise and decline.

Saliva samples were collected directly in marked tubes and stored at –20°C until assayed. Two separate samples of oral mucosa from each side of the cheek were scrubbed by cytological brushes into sampling tubes containing an RNA stabilizing solution (RNAlater, Sigma-Aldrich, St Louis, USA) and stored at –20°C until further analysis.

#### 2.2.2. Svalbard sampling conditions

**Figure 1:**
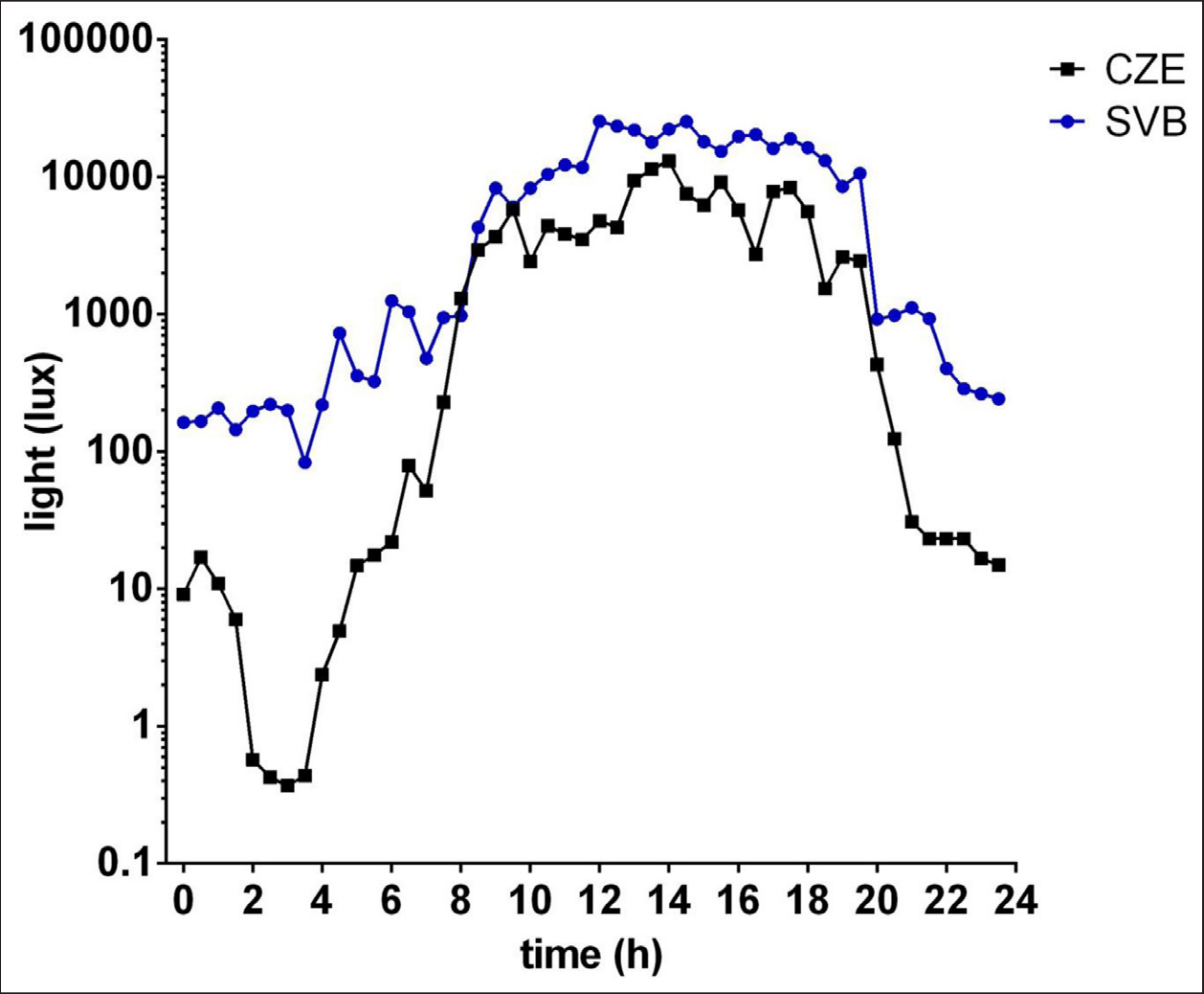
**The average light intensity in the Czech Republic (black line) and in Svalbard (blue line) during the 24 h of polar day.** The light intensity is plotted on logarithmic scale.

The expedition took place at Nostoc Field Station in Petuniabukta in July. Sun at this latitude (78°41′13″N 16°31′43″E) is present for 24 hours at a time from the 7th of May to the 24th of August. The perceived light intensity during the polar day was almost stable, but actigraphic measurement revealed variations of the peak light level comparing midday points. During the night time hours, the subjects slept in containers with a small window that limited light exposure, but the light intensity measured by actigraphy was higher compared to that in the Czech Republic (Figure 1). On the 13^th^ day of the expedition, the subjects were provided with the prepared kits with sample tubes and brushes and followed the same sample collection protocol as in the Czech Republic. The saliva samples were stored and transported on dry ice and the buccal scrubs were transported in cooling boxes. All samples were processed in the Czech Republic.

### 2.3. Activity, sleep/wake, and light intensity recording

Activity was recorded using a MotionWatch device (MotionWatch model 8; Cambridge Neurotechnology Ltd. UK). All subjects wore the MotionWatch devices on their non-dominant hands, and they were instructed to only take them off during baths or saunas. The device recorded movement and light intensity every 30 s, and the overall daily activity was expressed as a mean activity within 30-min intervals 24 h/day. The MotionWatch data was analyzed using MotionWare software (Cambridge Neurotechnology Ltd. UK). Sleep analysis was conducted with particular attention to sleep time, wake-up time, and sleep duration. Actigraphic sleep/wake data was aligned with a sleep diary, which helped to distinguish between motionless periods in a waking state and real sleep. This provided more accurate information about sleep onset and offset. The mean activity was analyzed with cosinor analysis from eight subjects (two subjects were excluded due to defective MotionWatch devices). We used the cosinor analysis for each of our subject separately and the results of cosinor analysis were presented as a mean of the calculated values.

### 2.4. Melatonin assay

Salivary melatonin concentrations were evaluated using a commercially available direct double-antibody radioimmunoassay kit (Bühlmann Laboratories. Allschvil, Switzerland) according to the manufacturer’s instructions. The melatonin concentration was expressed in pg/ml as the mean ± SEM of eight subjects (two subjects did not provide a sufficient amount of saliva in every sample). Individual melatonin profiles were analyzed by cosinor analysis, as described in Chapter 2.6. Amplitude, mesor, and acrophase were assessed for individual profiles.

### 2.5. Determination of clock gene expression by quantitative real-time polymerase chain reaction (RT-qPCR)

The mRNA was extracted from buccal scrubs using the Direct-Zol RNA MiniPrep (Zymo Research Corporation) and each participant’s full RNA sample was reverse-transcribed by High-Capacity cDNA Reverse Transcription Kit (LifeTechnologies, Applied biotechnologies) in 20 ul reaction incubated 10 min at 25°C, 120 min at 37°C, and 5 min at 85°C. The cDNA was then diluted 1:2 with RNase-free water, and 2 µl of diluents were used to determine gene expression in a 16 µl qPCR reaction. Each reaction also contained 10.3 µl of PCR-grade water, 3.1 µl of HOT FIREPol Probe qPCR Mix Plus (Solis BioDyne, Estonia), and 0.6 µl of TaqMan Gene Expression Human FAM-MGB assay (Life Technologies, CA, USA) specific for the following genes: *Period 1* (*PER1* NM 002616, cat. no. Hs01092603_m1), *aryl hydrocarbon receptor nuclear translocator-like* (*ARNTL*. syn. *BMAL1* NM 001178, cat. no. Hs00154147_m1), *nuclear receptor subfamily 1 group D member 1* (*NR1D1* NM 021724, cat. no. Hs00253876_m1), *beta-2-microglobulin* (*B2M* NM 004048, cat. no. Hs00187842_m1), and *glyceraldehyde-3-phosphate dehydrogenase* (*GADPH* NM 002046, cat. No. Hs99999905_m1). The qPCR reactions were performed in triplicate and amplified in sealed 384-well microplates on a LightCycler® 480 instrument (Roche Life Science, Indianapolis, IN, USA) using the following temperatures: initial denaturation at 95°C for 15 min, followed by 50 cycles consisting of denaturation at 95°C for 20 s and annealing/elongation at 60°C for 60 s. A negative control without the cDNA showed no amplification. As a positive control, a cDNA sample isolated from cultured human fibroblasts was used. The mean of the crossing point (Cp) was normalized to the geometrical Cp mean of *B2M* and *GADPH* housekeeping genes and then used for the analysis of relative gene expression using the ΔΔCT method [6].

### 2.6. Statistics

Activity data was plotted as a mean ± SEM in 30 min intervals bins for 24 h over the 12-day recording period. The activity profiles’ Czech and Svalbard measurements were compared using repeated-measure 2-way ANOVA with Bonferroni’s multiple comparison. Each of the activity profiles was analyzed with cosinor analysis, and the difference in acrophases among the groups was compared by paired Student’s t-test. Sleep analysis parameters were evaluated by paired Student’s t-test, while the differences between the Czech and Svalbard sleep parameters were analyzed by Wilcoxon’s test.

The data for melatonin levels at each time point was plotted as mean ± SEM for each group and compared by repeated-measure 2-way ANOVA with Bonferroni’s multiple comparison. The individual melatonin profiles were analyzed by cosinor analysis. The differences in acrophases and amplitude were evaluated by paired Student’s t-test, while the differences between the Czech and Svalbard measurements were analyzed by Wilcoxon’s test.

The data for clock gene expression was analyzed with cosinor analysis (see below) individually and in a group. The group results were expressed as mean ± SEM. The acrophase and amplitude among the groups were compared by paired Student’s t-test. The expression profiles among the groups were analyzed by 2-way ANOVA for repeated measures with Bonferroni’s multiple comparison.

Cosinor analysis: The data was fitted with two alternative regression models: either a horizontal line (null hypothesis) or a single cosine curve (alternative hypothesis) as defined by the equation Y = mesor + [amplitude*cos (2*π*(X-acrophase)/period)] with a constant period of 24 hours (Weissova et al. 2016). The analysis was done in Prism 8 software (GraphPad, La Jolla, USA).

## 3. Results

### 3.1. Actigraphic data

Using the actigraphic data, we performed nonparametric circadian analysis of rest/activity patterns and sleep/wake patterns for eight subjects (two subjects were excluded due to defective MotionWatch devices). We compared the data recorded in the Czech Republic before attending the Svalbard expedition with the data recorded in Svalbard.

#### 3.1.1. Activity

**Figure 2:**
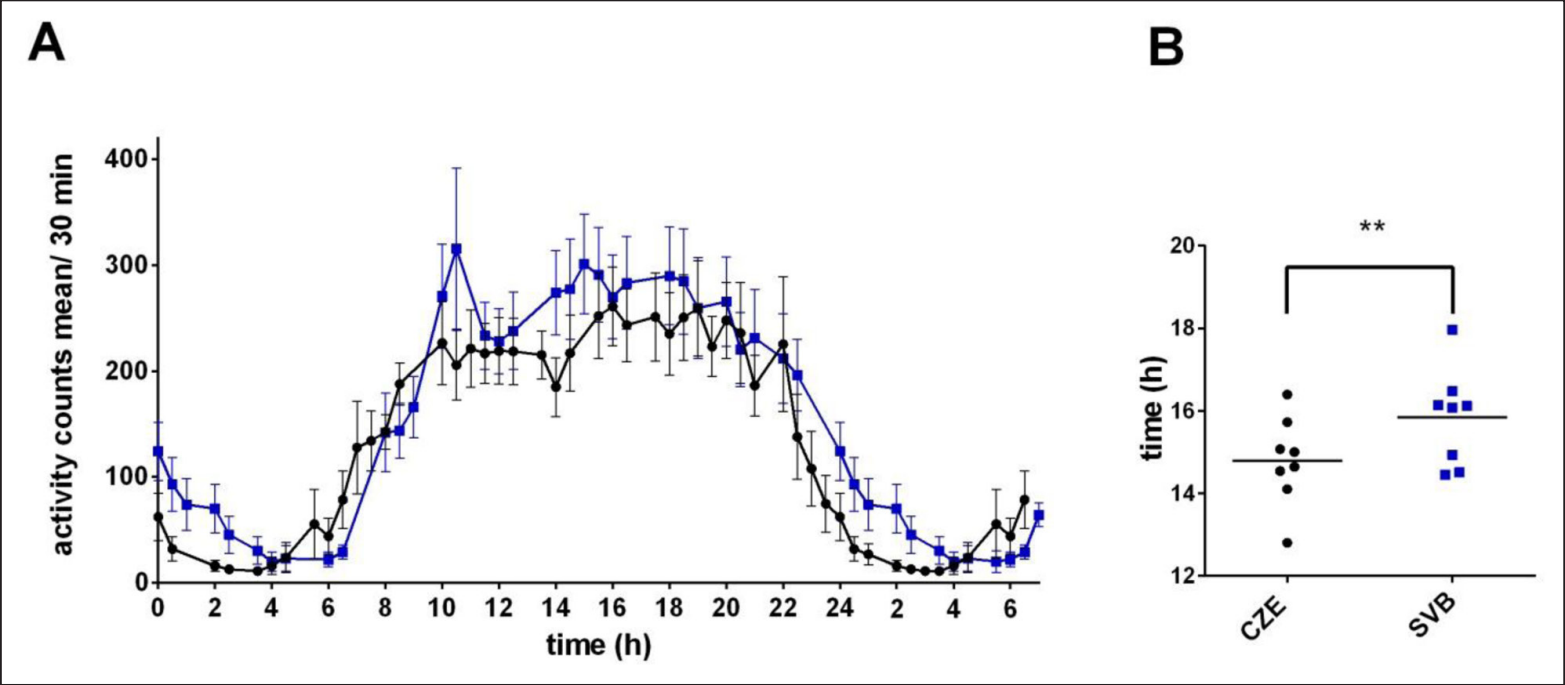
**Activity rhythms of subjects in Czech Republic (CZE) and in Svalbard (SVB). A)** Mean daily activity of eight subjects in the Czech Republic and in Svalbard. The activity was recorded by MotionWatch for 12 days and displayed as a mean ± SEM in 30 min bins over 24 h. For clarity, part of the day (00:00 to 7:00) was re-plotted. In the Czech Republic, the activity counts are represented by black dots and a black line, and in Svalbard by blue boxes and a blue line. **B)** Activity acrophase plotted for each subject. Black dots represent the acrophase for each subject in the Czech Republic; blue boxes present the acrophase in Svalbard.

The activity is presented as a mean in 30 min intervals throughout the day (Figure 2A). The repeated measures 2-way ANOVA using Bonferroni’s multiple comparison tests revealed a significant effect of time (F = 18.25; P < 0.0001), confirming the presence of daily variation in activity in both groups; however, there was no significant difference between the groups (F = 0.9007; P = 0.6902). Cosinor analysis followed by paired t-test identified a significant difference in acrophase between Czech and Svalbard circadian activity. Activity in Svalbard had been delayed 0.97 ± 0.1 h (P = 0.0021; Figure 2B).

#### 3.1.2. Sleep analysis

The analysis of sleep and nonparametric circadian parameters was performed by MotionWare software. We compared the times when participants fell asleep, the times at which they woke up, and total sleep duration.

**Figure 3:**
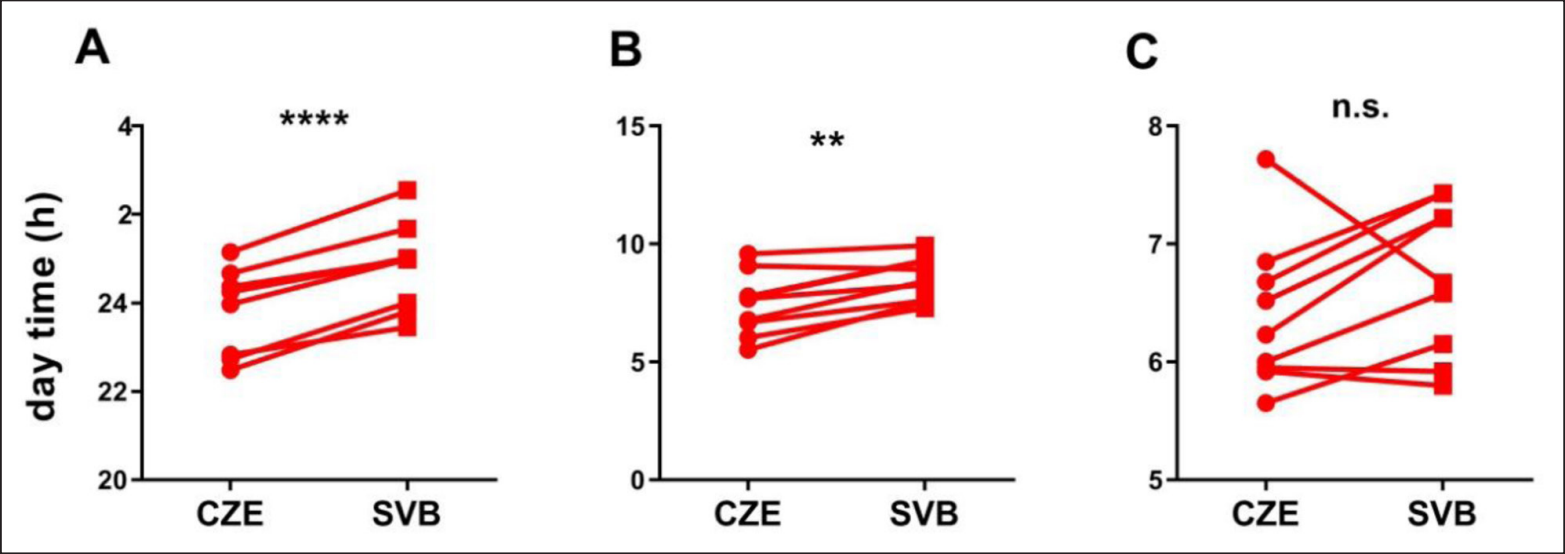
**Sleep parameters in the Czech Republic (CZE) and in Svalbard (SVB). A)** Fall-asleep time for each subject in the Czech Republic and in Svalbard. **B)** Wake-up time for each subject in the Czech Republic and in Svalbard. **C)** Sleep duration for each subject in the Czech Republic and Svalbard.

Student’s t-test revealed significantly later fall-asleep time in Svalbard (Czech Republic mean: 23.79 ± 0.98 h; Svalbard mean: 24.8 ± 1.03 h; P < 0.0005; Figure 3A). Similar results were observed for wake-up time means, for which the subjects’ mean wake times in Svalbard were significantly delayed as well (Czech Republic mean: 7.51 ± 1.4 h; Svalbard mean: 8.51 ± 0.97 h, P = 0.0056; Figure 3B). We did not find any significant difference in mean sleep duration (Czech Republic mean: 6.36 ± 0.67 h; Svalbard mean: 6.65 ± 0.65 h; Figure 3C).

### 3.2. Salivary melatonin level

**Figure 4:**
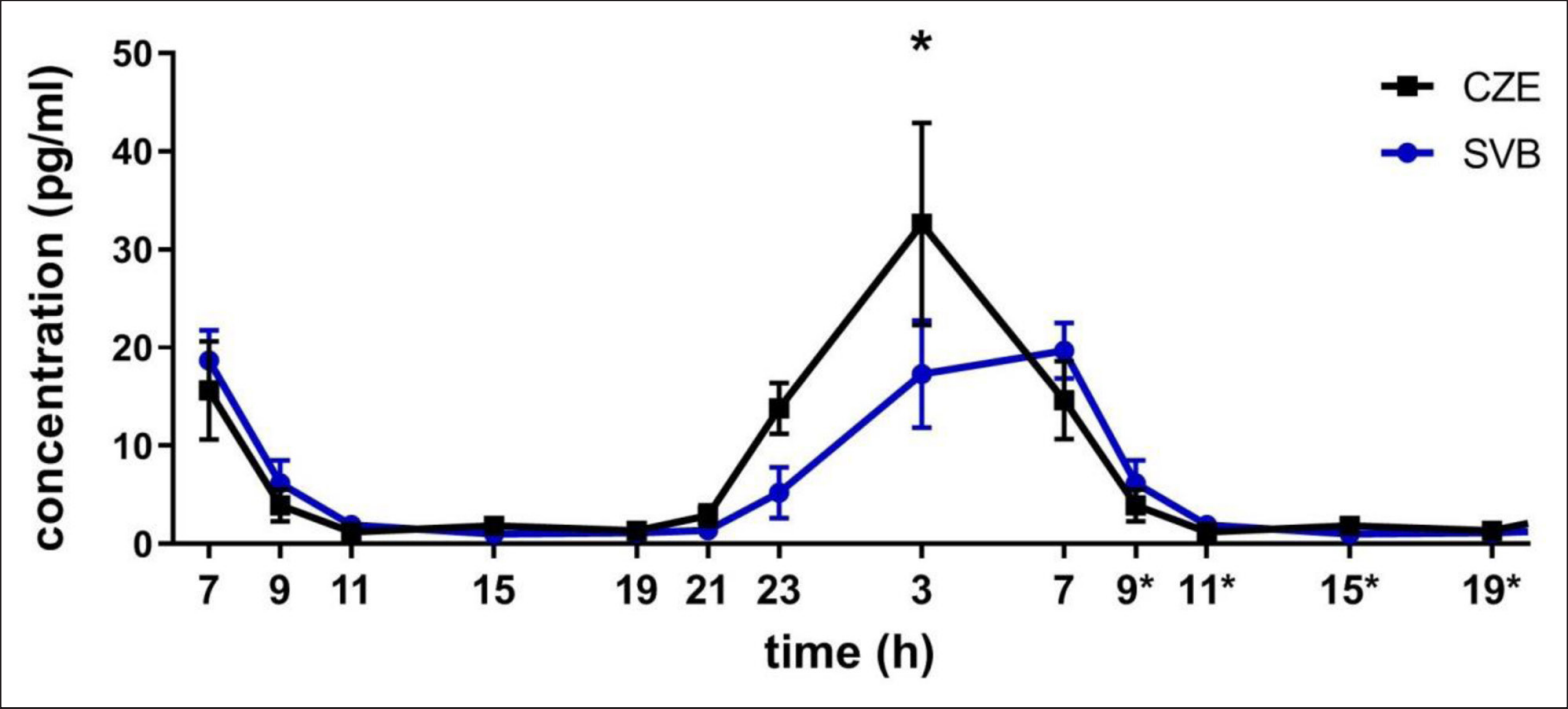
**Daily profiles of melatonin levels in saliva in the Czech Republic (CZE) and in Svalbard (SVB).** Melatonin levels were detected in saliva in 24 h profiles and expressed as mean ± SEM in pg/ml (n = 8). Black boxes and a black line represent melatonin levels in the Czech Republic and blue circles and blue lines represent melatonin level in Svalbard.

**Figure 5:**
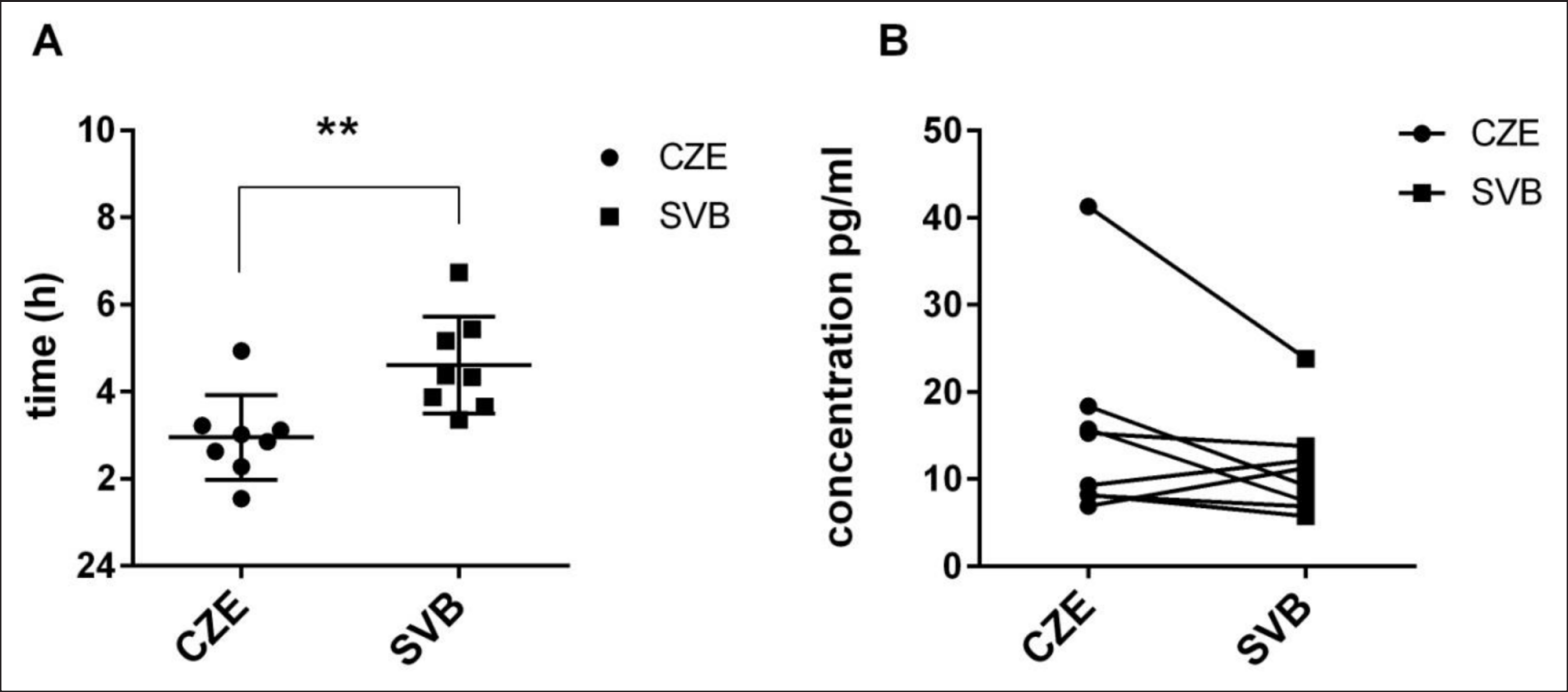
**Cosinor analysis in melatonin daily rhythm. A)** Melatonin acrophase plotted for each subject in the Czech Republic (black dots) and in Svalbard (blue boxes). **B)** Melatonin amplitude plotted for each subject in the Czech Republic (black dots) and in Svalbard (black boxes).

Melatonin levels were determined from saliva samples collected 9 times during the 24-h period. The daily melatonin is presented as a mean over the 32-h period with re-plotted values at 9*, 11*, 15*, and 19* hours. The repeated measures 2-way ANOVA using Bonferroni’s multiple comparison test revealed a significant effect of time (F = 12.5; P < 0.0001; Figure 4), which confirmed the daily variation in melatonin secretion in both groups. However, there was no significant difference between the samples collected in Svalbard and in the Czech Republic (F = 1.617; P = 0.0654). Multiple-comparison test revealed significant differences at 3 AM between the groups (P = 0.0355). Despite no significant difference between those two conditions, there was a significant difference in acrophase between melatonin rhythmicity in the Czech Republic and Svalbard (paired Student’s t-test; P = 0.0030; Figure 5A). The melatonin rhythm in Svalbard was delayed by about 1.666 ± 1.14 h. The comparison of amplitude between the samples collected in Svalbard and in the Czech Republic did not reveal significant differences due to a large SEM in the Czech time point (Figure 5B).

### 3.3. Clock gene expression in oral mucosa

**Figure 6:**
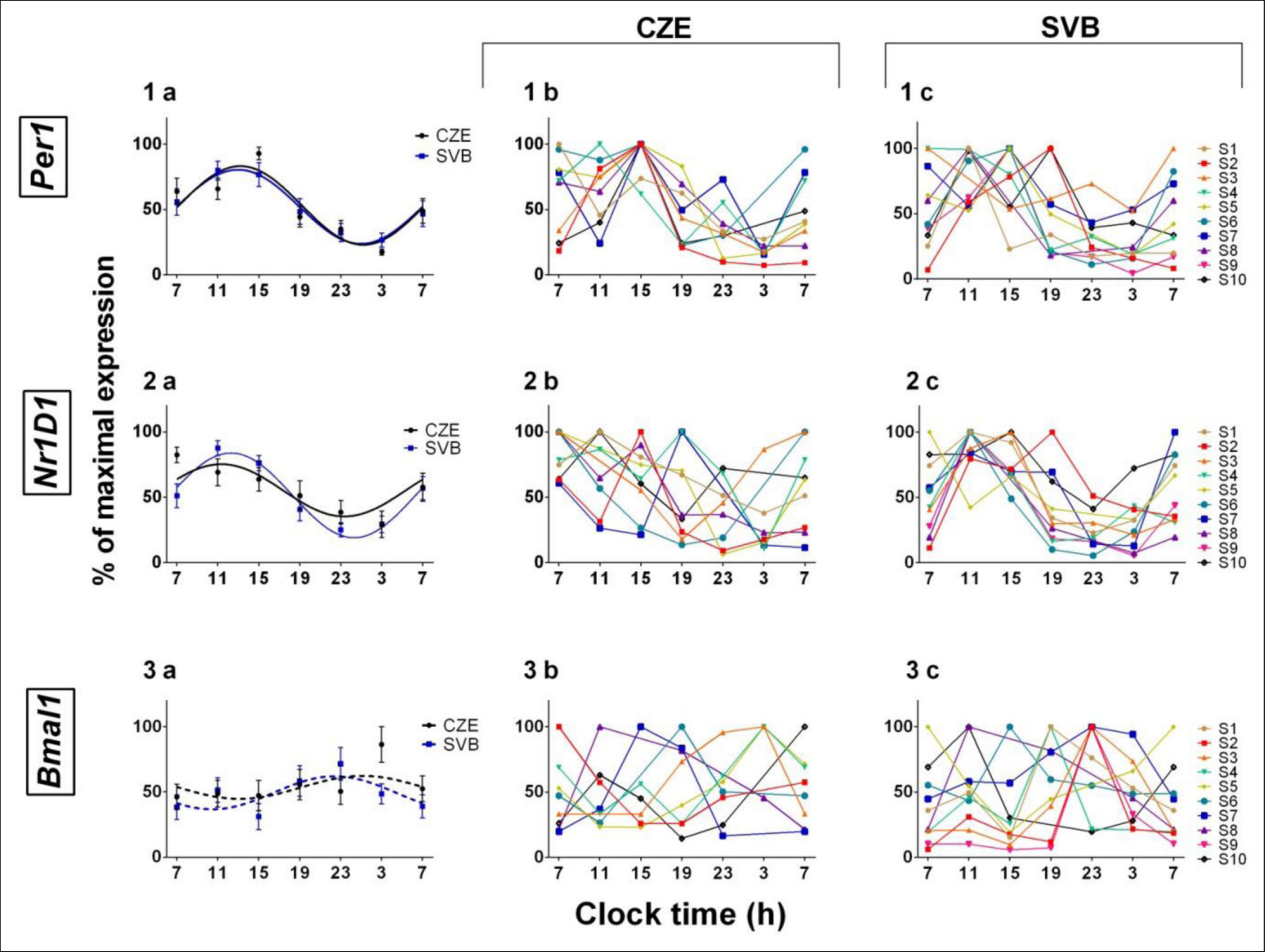
**Individual and group clock gene expression in oral mucosa.** 1) *Per1*, 2) *Nr1D1*, and 3) *Bmal1*. **a)** Cosinor analysis of the group means ± SEM 24-h rhythms in *Per1, Bmal1*, and *Nr1D1* gene expression. **b)** Individually plotted clock gene expression in the Czech Republic. **c)** Individually plotted clock gene expression in Svalbard. Relative gene expression is displayed in % of maximal value of its expression. Significant rhythms (P < 0.05 for *Per1, Nr1D1*) are indicated by a solid line; non-significant rhythms are indicated by a dotted line. The group averages are means ± SEM.

**Figure 7:**
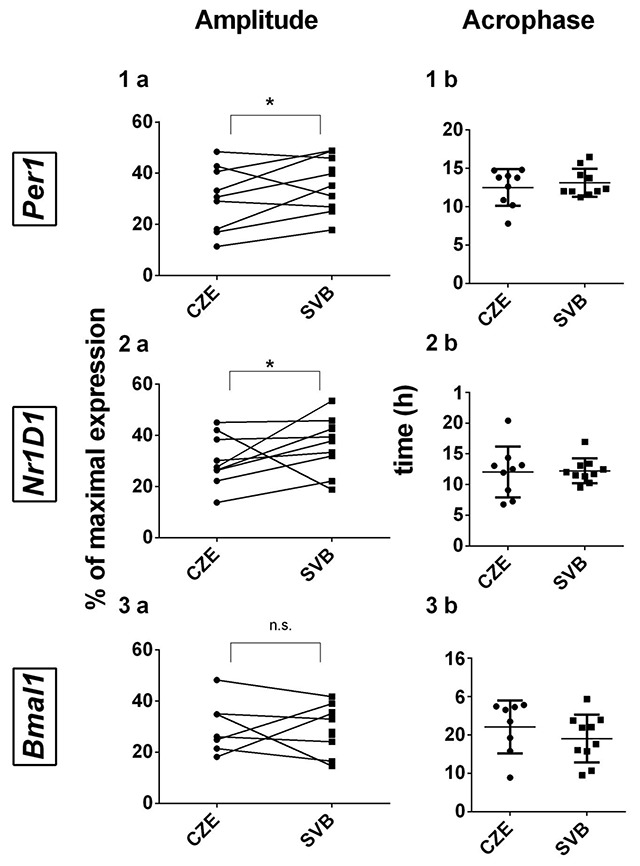
**The differences in amplitudes and acrophases for individual clock genes’ profiles in the Czech Republic and in Svalbard.** 1) Individually plotted amplitudes compared by paired Student’s t-test. 2) Individually plotted acrophases compared by paired Student’s t-test. **A)** *Per1*, **B)** *Bmal1*, and **C)** *Nr1D1*. The dots represent the values in the Czech Republic and the squares represent the values in Svalbard.

The *Per1, Nr1D1* and *Bmal1* expression profiles in oral mucosa were analyzed with cosinor analysis. The *Per1* and *Nr1D1* expressions showed significant circadian rhythmicity (Figure 6; 1a, 2a). Neither the Svalbard samples nor the Czech samples showed significant rhythmic expression in *Bmal1* gene expression (Figure 6, 3a). The repeated measures 2-way ANOVA with Bonferroni’s multiple comparison test revealed a significant effect of time (*Per1*: F = 12.5; P < 0.0001; *Nr1D1*: F = 11.08; P < 0.0001), which confirmed the presence of daily variation in gene expression in both groups. However, there was no significant difference between the groups (*Per1*: F = 0.966; P = 0.4527; *Nr1D1*: F = 1.050; P = 0.3989). The comparison of amplitude using paired Student’s t-test revealed a significant difference in *Per1* and in *Nr1D1* (*Per1*: P = 0.042; *Nr1D1*: P = 0.0244; Figure 7; 1a, 2a). However, there was no significant difference in acrophase, although the SD range was smaller in Svalbard (*Per1*- CZE: SD ± 3.13; SVB: SD ± 1.82; *Nr1D1*-CZE: SD ± 4.36; SVB: SD ± 2.03; *Bmal1*- CZE: SD ± 6.89; SVB: SD ± 6.26; Figure 7; 2a, 2b, 2c).

## 4. Discussion

The aim of this study was to examine the actual ability of the circadian clock to adapt to the polar day in real-life conditions. Each subject enrolled in the study was exposed to both study conditions: the Czech summertime and the Svalbard polar day. To our knowledge, there was only one study reporting such results with a similar study design. That study included only three subjects and was focused on different circadian parameters (oral temperature, self-reported fatigue, grip strength, heart rate, time of waking and getting up;) [1].

Our results suggest that circadian rhythmicity in the tested group of researchers differed based on the conditions in the Czech Republic and after two weeks in Svalbard. The activity rhythm was delayed in Svalbard, similarly to the salivary melatonin rhythm. The peripheral clock gene expression displayed higher amplitude and the acrophase standard deviation was lower. Comparing the mean overall activity between Svalbard and the Czech Republic, we did not observe any significant changes in its level. Sleep analysis confirmed that the majority of subjects postponed their wake-up and fall-asleep times without any significant changes in sleep duration. The mean salivary melatonin profiles displayed circadian rhythmic production in both conditions, but the night maximum in Svalbard was decreased and its rhythm was significantly delayed in the same manner as the activity. Delayed sleep/activity patterns and melatonin production during the polar day correspond with previous findings by other studies [2,3,4,5] and could be the result of the exposure to bright light until bedtime (Figure 1). Although the subjects slept in a facility with less light, the level of light during the night was higher than in the Czech Republic, which may provide some explanation for lowered melatonin levels during the night, most significantly at 3 AM. A comparison of within-subject profiles suggests that the lowered amplitude in Svalbard may be more general, and in the group analysis, it may be masked by the distinct individual phases of the melatonin rhythms.

To assess the circadian clock gene expression, we used oral mucosa samples collected every 4 hours. Oral mucosa samples provide a sufficient amount of RNA to analyze the circadian oscillation in oral mucosa cells [7,8]. Statistical analysis did not confirm the circadian rhythmicity of *Bmal1* expression. The low amplitude of *Bmal1* in samples of peripheral tissue has been shown in many other studies [8,9,10]. High inter-individual variability in phase and amplitude in our samples could further enhance the masking of the grouped rhythm. We observed that *Per1* and *Nr1D1* genes display strong circadian oscillation under both conditions, but surprisingly, the amplitude was significantly higher in the Svalbard samples and the acrophase standard deviation was lower. This might suggest a higher degree of synchronization of the circadian system in Svalbard. Increased amplitudes of *Per1* and *Nr1D1* were found even in individuals’ expression profiles. The smaller acrophase variability suggests that the synchronization in Svalbard could be better even between subjects.

In humans, the absence of or weakened photic zeitgeber may be compensated for by other zeitgebers, such as physical activity, social cues, regular mealtimes or drug intake [11,12,13]. A field study by Reinberg et al. (1984) did not show any significant alteration in circadian parameters of activity-rest rhythm, oral temperature, or fatigue rhythm after 63, 141, and 147 days in polar day conditions. The authors concluded that social synchronization might help to maintain the rhythmicity of subjects [1]. Other studies demonstrate that the circadian rhythmicity is stronger during constant light or darkness when the subjects keep their regular daily structures and suggest that a strict daily regime could very efficiently stabilize circadian rhythms [4,14]. On the contrary, Kennaway et al. reported free-running rhythms in melatonin and activity/rest rhythm. However, the authors discussed that the subjects in their study were encouraged to follow their individual sleep needs, and there was no pressure on them to work within time constraints [15].

Concerning our data, we hypothesize that in the Czech Republic, each of the subjects followed his or her subjective daily routine and they differed in their lifestyles. This could be reflected by higher variability in clock genes’ oscillation and melatonin rhythmicity as well. Regular social interaction among the subjects, shared mealtimes, and strict sleep-wake and working schedules in Svalbard could entrain the circadian rhythmicity even during extreme photoperiods such as a polar day. Alternatively, the slightly higher level of physical activity in Svalbard (see Figure 2) could further strengthen the synchronization of the peripheral clocks [16].

## 5. Conclusion

In general, circadian rhythms in activity (as a behavioral circadian marker), melatonin (as an indirect circadian marker), and peripheral clock gene expression (as a direct circadian marker) were slightly altered, but all of the subjects maintained circadian patterns in those studied parameters. Light-sensitive melatonin production was delayed by late-night light exposure; late-night illumination had the same effect on activity pattern, but the rhythm stayed consistent. A similar effect was observed in the peripheral clock genes’ expression; however, the rhythm was more pronounced. The delay in circadian clock-driven parameters such as melatonin production and the sleep/wake rhythm might result from the late night light exposure and Svalbard expedition work schedule rather than reflect a delay in the inner circadian clock. We hypothesize that the clock stayed fully synchronized under the conditions of the polar day due to the shared schedule between participants, particularly the mealtimes, which would also lead to strengthened social interactions. These factors have a cumulative positive effect on circadian rhythm synchronization.
